# *EDN1* Lys198Asn is associated with diabetic retinopathy in type 2 diabetes

**Published:** 2008-09-15

**Authors:** Haitao Li, Janice W.C. Louey, Kwong Wai Choy, David T.L. Liu, Wai Man Chan, Yiu Man Chan, Nicholas S.K. Fung, Bao Jian Fan, Larry Baum, Juliana C.N. Chan, Dennis S.C. Lam, Chi Pui Pang

**Affiliations:** 1 Department of Ophthalmology and Visual Sciences, The Chinese University of Hong Kong, Hong Kong, China; 2 Faculty of Medicine, Dentistry and Health Sciences, The University of Melbourne, Australia; 3 Department of Obstetrics and Gynecology, The Chinese University of Hong Kong, Hong Kong, China; 4 Department of Ophthalmology, Hong Kong Sanatorium and Hospital, Hong Kong, China; 5 Department of Ophthalmology, Harvard Medical School, Massachusetts Eye and Ear Infirmary, Boston, MA; 6 Department of Medicine and Therapeutics, The Chinese University of Hong Kong, Hong Kong, China

## Abstract

**Purpose:**

We tested the hypothesis that genetic variants in vasoactive and angiogenic factors regulating the retina vasculature contribute to the development of diabetic retinopathy (DR).

**Methods:**

A case-control study was performed to study the genetic association between DR and polymorphic variants of *EDN1* (Lys198Asn), *LTA* (IVS1–80C>A, IVS1–206G>C, IVS1–252A>G), *eNOS* (Glu298Asp), and *ITGA2* (*BgI II)* in a Chinese population with type 2 diabetes mellitus. A well defined population with type 2 diabetes, consisting of 127 controls and 216 DR patients, was recruited.

**Results:**

A higher frequency of the Asn/Asn genotype of *EDN1* was found in individuals with at least 10 years of diabetes and no retinopathy (controls) compared with DR patients with any duration of diabetes (DR: 2.3%; control: 11.0%; p=0.0002). The Asn allele was also more frequent in controls than DR patients (DR: 16.4%; control: 29.5%; p=0.007). Multiple logistic regression analysis showed that the Asn/Asn genotype was the factor most significantly associated with reduced risk of DR (odds ratio=0.19; 95% CI: 0.07-0.53; p=0.002) and with late onset of diabetes (Asn/Asn: 59 years; Lys/Lys + Lys/Asn: 53 years; p=0.02). Moreover, the Lys/Lys genotype was more common among patients with nonproliferative (75.7%) than proliferative DR (56.9%; p=0.008). The distributions of Lys198Asn alleles in hypertension did not differ from normotensive subjects. No associations between DR and polymorphisms of *LTA*, *eNOS*, or *ITGA2* were detected, and there were no detectable gene-gene or gene-environmental interactions among the polymorphisms.

**Conclusions:**

The Asn/Asn genotype of *EDN1* was associated with a reduced risk of DR and with delayed onset of type 2 diabetes.

## Introduction

Diabetic retinopathy (DR), a leading cause of blindness worldwide, is a microvascular complication of diabetes characterized by increased vascular permeability and hemostatic abnormalities which can eventually lead to vascular occlusion in spite of antidiabetic treatment and result in retinal nonperfusion and neovascularization [1]. DR is associated with disorders in the nitric oxide (NO) pathway, including impaired NO-mediated vasodilation, increased oxidative and nitrative stress, dysregulation of NO synthase isoforms, and endothelial NO synthase uncoupling [2]. Clinically, DR can be classified into two major categories: early and advanced. Early stage of DR, also called nonproliferative diabetic retinopathy (NPDR), is characterized by edema, leakage of fluid, and limited blood flow into the eye. But NPDR has no abnormal neovascularization. The advanced stage of DR, or proliferative diabetic retinopathy (PDR), involves neovascularization and fibrous tissue formation [3].

Over 30 candidate genes involved in different metabolic mechanisms and functional pathways have been reported to be associated with DR [4,5]. However, only a fraction of them have shown consistent associations with occurrence of DR or its severity in different studies [4,5]. The (CA)_n_ microsatellite marker at the 5’-end of the aldose reductase (*AR2*) gene is the most frequently reported polymorphism that is associated with DR [4]. Results of our previous study on Chinese DR patients showed that the z-2 allele of the 5′-(CA)_n_ polymorphism was independently associated with DR in type 2 diabetes [6]. Genetic studies have been conducted for vasoactive and angiogenic factors to explore their contributions to DR, such as endothelial nitric oxide synthase (*eNOS*), lymphotoxin-a (*LTA*), integrin alpha-2 (*ITGA2*), angiotensin converting enzyme (*ACE*), vascular endothelial growth factor (*VEGF*), intercellular adhesion molecule 1 *(ICAM* or *CD45*), β3-adrenergic receptor gene *(ADRB3)*, and endothelin-1 (*EDN1*) [4,5]. However, results for some of these genes, such as *ACE* and *CD45*, have been inconsistent in various ethnic groups and different study populations. Discrepancies among these studies are likely due to variations in case definition, sample sizes, and medical conditions of control subjects. Some studies selected normal individuals as controls instead of diabetic subjects without DR. Thus, the comparisons were only between normal individuals and diabetic individuals, which may lead to identification of genetic variants associated with vulnerability to diabetes mellitus (DM) rather than to DR [7].

In this study, we focused on a well-defined population with type 2 diabetes. Only patients without DR but with DM for at least 10 years were selected as controls. We hypothesized that genetic variants in vasoactive and angiogenic factors which regulate the retina vasculature might contribute to the development of retinopathy. We chose polymorphisms previously reported to have positive association with DR, but none have been evaluated in a Chinese DM population. The Lys198Asn polymorphism of *EDN1* was also studied because it is associated with hypertension, which is a risk factor of DR [8-11]. We examined six polymorphisms in four genes, including two vasoactive genes, *EDN1* and *eNOS*, and two hemodynamic blood flow related genes, *LTA* and *ITGA2.* They have been reported to be associated with vascular diseases in different populations.

## Methods

### Study subjects

Unrelated participants with type 2 noninsulin-dependent diabetes mellitus (NIDDM) were recruited from the Eye Clinic of the Prince of Wales Hospital, Hong Kong. Diagnosis of type 2 diabetes was based on WHO criteria [12]. This study was performed in accordance with the ethics standards set by the Declaration of Helsinki. Approval for use of human subjects was obtained from the New Territories East Cluster, Hong Kong, clinical research ethics committee. Informed consent was obtained from the participants after explanation of the nature and possible consequences of the study. DR was diagnosed in a masked manner by independent ophthalmologists by direct ophthalmoscope through dilated pupils. Patients with no signs of DR and with known DM duration <10 years were excluded. Patients without DR but with diabetes duration ≥10 years were designated as control subjects (DM). Diabetic patients with DR were defined as case subjects (DR) either of the NPDR or PDR subtype according to Early Treatment Diabetic Retinopathy Study (ETDRS) criteria [13]. Documented information included age, sex, age at onset of diabetes, duration of diabetes, fast plasma glucose and hemoglobin A_1_c (HbA_1_c) level, age at onset of DR, family history, and treatment details. A smoker was defined as someone who smoked at least five cigarettes daily for more than one year. Onset of DR was defined as the first ever documentation of the clinical evidence of DR on the chart. Hyperlipidemia was diagnosed on patients with either elevated total cholesterol (>6.2 mM) or elevated triglycerides (>1.7 mM) requiring either lifestyle modification or pharmacological intervention. Renal failure was defined as persistent and irreversible derangement of the renal function test, typically creatinine >80 μM upon consecutive measurements.

After exclusion of non-DR patients having DM duration of fewer than 10 years, 343 patients were enrolled, consisting of 127 controls and 216 DR patients. Using the allele distributions in non-DR patients, we calculated the statistical power at 80% with a Bonferroni correction significance level of 0.0083, where six comparisons were made, and α, before correction was 0.05. Thus the corrected level, α ÷ 6 is 0.083 (two-sided) to detect an allele odds ratio of at least 2.2 for eNOS or 1.8 for the other five polymorphisms. DR patients were further divided into NPDR (n=144) and PDR (n=72). DR group showed a significantly higher level of HbA_1_c (7.35±1.36% versus 6.9±1.91%, p=0.046) and a higher percentage of patients who underwent insulin treatment (11.5% versus 2.4%, p<0.00001) than control group (Table 1). The PDR group was slightly younger than NPDR and had earlier onset of diabetes. Diabetic macular edema was more common in PDR than in NPDR.

**Table 1:** Clinical and metabolic characteristics of patients with type 2 diabetes

**Characteristic**	**DM (n=127)**	**All DR (n=216)**	**NPDR (n=144)**	**PDR (n=72)**
Age (years)	68.8±11.2	66.8±10.4	69.1±9.4	62.7±11.0^4,5^
Gender (% male)	60.6	60.6	59.4	62.5
Age at onset of DM (years)	53.8±11.9	53.0±10.8	55.4±10.4	48.4±10.0^6,7^
Duration of diabetes (years)	14.7±4.8	13.9±8.3	13.7±8.0	14.4±8.8
Age at onset of DR (years)	None	62.1±10.2	65.6±9.4	56.8±9.9
Gap between DR and DM onset ages (years)	None	8.4±8.8	9.1±7.6	7.3±11.1
Duration of DR (years)	None	4.9±3.8	4.6±3.6	5.6±4.1
HbA1c (%)	6.90±0.91	7.35±1.39^1^	7.53±1.56^3^	6.98±0.94
Fasting plasma glucose (mM)	8.24±1.98	8.26±2.83	8.09±2.86	8.81±2.89
Insulin use (% of patients)	2.4	11.5^2^	8.3	18.1^8^
Family history of DM (% of patients)	17.7	25.3	22.5	29.2
Hypertension (% of patients)	68.8	67.1	66	69.4
Hyperlipidemia (% of patients)	26.8	24.8	24.5	26.4
Renal failure (% of patients)	0.8	4.1	3.5	5.6
Current smoking (% of patients)	9.2	11.1	11.1	11.1
Diabetic macular edema (% of patients)	None	31.9	26.4	43.1^9^

The table showed the comparisons of the clinical and metabolic characteristics among diabetes mellitus controls (DM) and diabetic retinopathy (DR) and DR subtypes. All p values were computed by χ2 or student t-tests. DR group showed a significantly higher level of HbA1c and higher percentage of patients receiving insulin treatment than controls (1: p=0.046; 2: p<0.00001). By Tukey’s test for Post Hoc multiple comparisons (the Bonferroni corrected significance level was 0.025=0.05÷2), the significant difference of HbA1c level was only found between nonproliferative diabetic retinopathy (NPDR) and controls (2: p=0.001) and the significant higher percentage of the patients receiving insulin treatment was only due to proliferative diabetic retinopathy (PDR) group (8: p=0.00009). The PDR group was slightly younger than NPDR (4: p=0.001) and controls (5: p=0.00005) and with earlier onset of diabetes than other two groups (6: p=0.0008; 7: p=0.000003). The presence of diabetic macular edema was evident in PDR compared to NPDR (9: p=0.001).

### Genotyping

The whole blood specimens (5 ml) from all the patients were collected in EDTA tube and stored at -20 °C for fewer than two months’ storage. Genomic DNA was extracted from whole blood using the Qiamp kit (Qiagen, Hilden, Germany) and stored at −20 °C for fewer than two months before analysis. The polymorphisms of *EDN1* (Lys198Asn or rs5370) and *LTA* (IVS1–80C>A, IVS1–206G>C, and IVS1–252A>G) were detected by polymerase chain reaction (PCR) and direct DNA sequencing. PCR was performed in a final volume of 25 μl containing 2.5 μl of 10X PCR buffer (Invitrogen^TM^ Life Technology, Carlsbad, CA), 0.3 μl of each primer (*EDN1*: forward 5’- CTT TTG CCA AAG GGT GAT TT-3’ and reverse 5’- AGG GTG GAG AGT GCA GAG TC-3’; *LTA*: forward 5’- TCC TGC CCC ATC TCC TTG G-3’ and reverse 5’-AGA GAG AGA GAC AGT GAG CGG G-3'), 0.5 μl of 10 mM dNTP, 1 μl of 50 mM MgCl_2,_ 0.5 U AmpliTaq® DNA Polymerase (Applied Biosystems, Foster City, CA), and 10 ng of DNA. After the initial denaturation at 94 °C for 3 min, 35 cycles were conducted: 94 °C for 30 s, annealing for 30 s (*EDN1*: 54 °C; *LTA*: 58 °C), and 72 °C for 30 s. The final extension lasted for 10 min at 72 °C. Sequencing was performed using a standard protocol on an automated 3130X DNA sequencer (Applied Biosystems) [14]. Sequence data were analyzed on computer (Chromas ver. 2.13; Technelysium Pty Ltd., Tewantin, QLD, Australia) and compared with published genes sequences from Ensembl. All rare variants detected were confirmed by bidirectional sequencing.

Polymorphisms in *eNOS* (894G>T) and *ITGA2* (IVS8–1059T>C) were analyzed by PCR followed by BanII and BgIII restriction analysis respectively [15,16]. To cross-check the genotyping results, we randomly selected one-fourth of the PCR products for direct sequencing. Complete matching of results was obtained.

### Statistical analysis

Differences in genotype distribution and consistency with Hardy–Weinberg equilibrium were tested by χ^2^ test. Continuous clinical data (age, age at onset of diabetes, duration of diabetes, and HbA_1_c) were compared by independent Student *t* test, and categorical clinical data were compared using the χ^2^ test or the Fisher’s exact test. Tukey’s test was used for multiple Post Hoc comparisons, and the Bonferroni method was used for multiple comparison adjustment. To assess the role of gene polymorphisms and search for gene-gene and gene-environmental interactions, we built logistic regression models using various polymorphisms and clinical parameters. Disease status was set as the dependent variable (DR=1; control=0), and gene polymorphisms and environmental factors as independent variables. A stepwise regression approach was used to optimize the analysis. SPSS for Windows, standard version 11.5 (SPSS, Chicago, IL), was used. Statistical significance was defined as p<0.05.

## Results

The distributions of the six polymorphisms of *EDN1*, *eNOS*, *LTA*, and *ITGA2* in both case and control groups were under Hardy–Weinberg equilibrium. A significantly higher frequency of the *EDN1* Asn/Asn genotype was found in controls than in DR patients (11% versus 2.3%, p=0.0002, Bonferroni corrected significance level 0.0083=0.05÷6, Table 2). The Asn allele frequency was also significantly higher in controls than in the DR group (29.5% versus 16.4%, p=0.007, Bonferroni corrected significance level 0.0083). For genotype or allele distributions of *eNOS*, *IGTA2,* and *LTA* polymorphisms, there was no significant difference between DM subjects with or without DR (Table 2).

**Table 2:** Genotype and allele frequencies in groups with and without retinopathy

**Polymorphism**	**Genotype distribution (%)**				**Allele distribution (%)**			
		**DM (n=127)**	**DR subtypes**			**DM (n=254)**	**DR subtypes**	
			**NPDR (n=144)**	**PDR (n=72)**			**NPDR (n=288)**	**PDR (n=144)**
*EDN1* Lys198Asn	Lys/Lys	66 (52.0)	109 (75.7)	41 (56.9)	Lys	179 (70.5)	249 (86.5)	112 (77.8)
	Lys/Asn	47 (37.0)	31 (21.5)	30 (41.7)	Asn	75 (29.5)	39 (13.5)	32 (22.2)
	Asn/Asn	14 (11.0)	4 (2.8)	1 (1.4)				
	p value	all DR versus DM: 0.0002				all DR versus DM: 0.007		
		NPDR versus DM: 0.0001				NPDR versus DM: 0.00005		
		PDR versus NPDR: 0.008						
*eNOS* Glu298Asp	Glu/Glu	98 (77.2)	110 (76.9)	55 (76.4)	Glu	224 (88.2)	249 (87.1)	127 (88.2)
	Glu/Asp	28 (22.0)	29 (20.3)	17 (23.6)	Asp	30 (11.8)	39 (12.9)	17 (11.8)
	Asp/Asp	1 (0.8)	4 (2.8)	0 (0)				
*ITGA2 *BgI II	BgI II (-/-)	65 (51.2)	76 (52.8)	33 (45.8)	-	184 (72.4)	211(73.3)	100 (69.4)
	BgI II (-/+)	54 (42.5)	59 (41.0)	34 (47.2)	+	70 (27.6)	77 (26.7)	44 (30.6)
	BgI II (+/+)	8 (6.3)	9 (6.3)	5 (6.9 )				
*LTA* IVS1-80C>A	CC	64 (50.4)	76 (53.1)	31 (43.7)	C	174 (68.5)	210 (73.4)	94 (66.2)
	CA	46 (36.2)	58 (40.6)	32 (45.1)	A	80 (31.5)	76 (26.6)	48 (33.8)
	AA	17 (13.4)	9 (6.3)	8 (11.3)				
*LTA* IVS1-252A>G	AA	31 (24.4)	30 (20.8)	19 (26.4)	A	127 (50.0)	134 (46.5)	71 (49.3)
	GA	65 (51.2)	74 (51.4)	33 (45.8)	G	127 (50.0)	154 (53.2)	73 (50.7)
	GG	31 (24.4)	40 (27.8)	20 (27.8)				
*LTA *IVS1-206G>C	GG	63 (49.6)	76 (52.8)	34 (47.2)	G	173 (68.1)	210 (72.9)	101 (70.1)
	GC	47 (37.0)	58 (40.3)	33 (45.8)	C	81 (31.9)	78 (27.1)	43 (29.9)
	CC	17(13.4)	10 (6.9)	5 (6.9)				

The genotype and allele frequency distributions are shown for diabetic retinopathy (DR) and controls (diabetes mellitus without diabetic retinopathy, DM). Only the *endothelin-1 (EDN1)* Lys198Asn genotype and its allele distributions showed a statistically significant difference between DR patients and controls at the Bonferroni corrected significance level of 0.0083=0.05÷6. For comparing DR subtypes and DM distributions of only the *EDN1* Lys198Asn polymorphism, the Bonferroni corrected significance level was 0.017=0.05÷3.

For the *EDN1* polymorphism, we compared DM controls and DR subtypes. NPDR patients had a higher frequency of Lys/Lys than either DM controls or PDR patients (75.7% versus either 56.9% or 52.0% with p=0.0001 and 0.008, respectively, Bonferroni corrected significance level 0.017 = 0.05 ÷ 3), but PDR did not differ from DM controls (p>Bonferroni corrected significance level 0.017). The Asn allele frequency was also significantly higher in controls than in NPDR (29.5% versus 13.5%; p=0.00005, Bonferroni corrected significance level 0.017). However, allele frequencies did not significantly differ among DR subtypes.

Multivariable logistic regression analysis showed that *EDN1* Asn/Asn was an independent protective factor for DR after adjustment for age, age at onset of diabetes and insulin therapy. The Odds ratio (OR) was 0.19 with 95% confidence interval (CI) ranging from 0.07 to 0.53 (p=0.002; Table 3). The difference of Lys198Asn genotype distributions between NPDR and PDR was not significant after adjusting for the age at onset of diabetes (p>0.05). But age at onset of diabetes was an independent factor associated with the PDR phenotype (OR=0.94; 95% CI: 0.91–0.97; p=0.00001). We also found no gene-gene or gene-environmental factor interaction in DM samples (p>0.05). The age of DM onset of patients with the Asn/Asn genotype was about six years later than patients with other genotypes (p=0.02; Table 4). Genotype distributions of Lys198Asn were not different between hypertensive and nonhypertensive subjects (Table 4).

**Table 3:** Odds ratio adjusted by multivariable logistic regression for the association with diabetic retinopathy in patients with type 2 diabetes

**Factors**	**Adjusted OR**	**Adjusted p value**
DM versus DR		
*EDN1*: Asn/Asn versus Lys/Lys+Lys/Asn	0.19 (0.07-0.53)	0.002
NPDR versus PDR		
Age at onset of diabetes	0.94 (0.91-0.97)	0.00001

The relationship between diabetic retinopathy (DR) and its affected factors were tested by multivariable logistic regression analysis. The presence of DR was regarded as the dependent variable, and independent variables included genotype of *EDN1, eNOS, ITGA*, and *LTA*, gender, age, age at onset of diabetes, HbA1c, therapy of insulin, hypertension, and hyperlipidemia. Only items with p values <0.05 were listed. After adjustment for age, age at onset of diabetes and insulin therapy, etc, *EDN1* Asn/Asn was the only independent protective factor for DR and younger onset age of diabetes was a significant risk factor for nonproliferative diabetic retinopathy (NPDR) progressing to proliferative diabetic retinopathy (PDR).

**Table 4:** Relationship between *EDN1*genotype, age of diabetes mellitus onset, and hypertension

**Factors**	**Lys/Lys**	**Lys/Asn**	**Asn/Asn**	**p value**
Number of patients	216	108	19	
Age of diabetes mellitus onset	53.1+/-11.1	53.0+/-11.6	59.0+/-11.6	0.02
Hypertensive	143 (61.9%)	74 (32.0%)	14 (6.0%)	0.79
Nonhypertensive	73 (65.2%)	34 (30.4%)	5 (4.5%)	

The onset age of diabetes mellitus (DM) and hypertension frequency were compared among different *EDN1* genotypes in all diabetic patients. The age of DM onset of patients with the Asn/Asn genotype was about six years later than patients with other genotypes (p=0.02) and Lys198Asn genotype distributions were not different between hypertensive and nonhypertensive subjects (p=0.79).

## Discussion

The vasodilator NO and vasoconstrictor endothelin-1 (ET-1) effectively determine the tone of blood vessels [2,17]. Changes in blood flow contribute to early diabetic microangiopathy in DR [18]. Genetic studies on *eNO*S and risk to DR have shown inconsistent results, and no association between polymorphisms in *EDN1* and DR has been identified [19-22]. LTA and ITGA2 are important molecules involved in lymphocyte proliferation and platelet adhesion to subendothelial tissues. They are essential for thrombus formation and contribute to tissue ischemia and activation of neovascularization in DR. The BgIII polymorphism of *ITGA2* has been shown to be strongly associated with DR in the Caucasian and Japanese populations [16,23]. There are also ethnic differences in the genetic risk of DR. *LTA* IVS1–252A>G has been found to be associated with DR in Caucasians but not in Japanese [7,24].

Our study is the first to investigate the *EDN1* Lys198Asn polymorphism in a diabetic population. The Asn/Asn frequency was reported to be 6.7% in the Chinese population (HapMap project). We found that the Asn/Asn genotype was higher in DM controls (11.0%) than in DR subjects (2.3%), suggesting a protective role against DR in general. In addition, Asn/Asn was associated with an older age of onset of diabetes, on average a six-year delay (Table 4). Therefore, Asn/Asn might delay DM and protect against progression of DM to NPDR. The Asn/Asn genotype was not associated with hypertension in our DM subjects, indicating that Asn/Asn might not modulate the risk of DR via an effect on hypertension. The frequency of Lys/Lys in PDR (56.9%) was closer to that in DM controls (52.0%; p>0.017) than in NPDR (75.7%; p<0.017). This implies that the pathological mechanisms that lead to NPDR might be different from those in PDR. Lys/Lys might contribute a role in the early breakdown of the blood retinal barrier (as may be the case in NPDR) rather than vascular occlusion and neovascularization (in the case of PDR).

In this study, we focused on a clinically well defined diabetic population to explore the role of genetic factors in DR progression. Each member of the control group lacked DR but had a ≥10-year history of diabetes. According to the Wisconsin epidemiologic study of diabetic retinopathy (WESDR), the 10-year incidence of DR was almost twice as high as the four year incidence of DR in older onset diabetic subjects (67% versus 34%). In insulin-dependent individuals diagnosed with diabetes before the age of 30 years, the incidence of DR increased from 59% over 4 years to 89% over 10 years, and maintained a similar incidence of 86% over 14 years [25-27]. Thus, a 10-year duration of DM is a reasonable criterion for selecting controls for a DR genetic study. Diabetes duration and glycemic control are major determinants of the development of DR. Our results also showed that subjects who were younger at DM onset had a higher risk of PDR than did subjects with later onset of DM (OR=0.94; p=0.00001; Table 3).

ET-1 is implicated in some vascular diseases, including the pathology of DR [28]. *EDN1* has also been identified in an early gestation human eye cDNA library and might be important for eye development [29]. The pathogenic significance of the association of *EDN1* Lys198Asn with DM and DR remains unknown. Some studies suggested that the Asn allele is associated with increased plasma concentrations of ET-1 [9,10]. However, ET-1 levels in vitreous and aqueous from DR patients were only elevated at the advanced stage of DR (PDR), but decreased in the early stage (NPDR) [17,30]. ET-1 is synthesized from a 212-amino-acid precursor protein (preproET-1) through multiple proteolytic steps. In the first step, preproET-1 is cleaved by signal peptidase, resulting in the formation of proET-1. ProET-1 is then cleaved at the paired dibasic amino acids by a furin-like enzyme to give rise to 38-aminoacid big ET-1 or other intermediates. Big ET-1 is subsequently cleaved at Trp73-Val74 by another endopeptidase, endothelin converting enzyme, resulting in the production of mature ET-1 [31]. A functional in vitro expression study of *EDN1* Lys198Asn found that neither ET-1 (bioactive form) nor big ET-1 (intermediate polypeptide) levels in the culture supernatant of the Asn-type transfected cells were significantly changed compared to those of the Lys-type transfected cells in three different cell lines: COS1 cells, 293 cells and human umbilical vein endothelial cells (HUVECs) [32]. Lys198Asn is located near the carboxyl terminal region, which is removed from prepro-ET-1 by the proteolytic action of the furin-like enzyme during the processing of ET-1 [10,33]. This polymorphism may affect the processing of preproET-1 to mature ET-1 rather than modifying the gene expression or the stability of the mRNA. So far there is no data on the functional consequence of the Lys198Asn polymorphism on preproET-1. We hypothesized that the protective effects of 198Asn on diabetes and DR might not exert direct influence on ET-1 production. It could have an impact on the processing of ET-1 or influence linkage disequilibrium with other polymorphisms that affect the production or conformation of the protein. Alterations in ET-1 level or function might affect the response to damage from hyperglycemia and hypoxia in vascular endothelial and other cell types. Another *EDN1* polymorphism, a dinucleotide repeat in the 5′-untranslated region, did not have a major impact on DR [19].

In conclusion, we identified the Asn/Asn genotype of *EDN1* as a genetic factor for delayed onset of DM and reduced risk of DR in type 2 DM patients. Further studies in other DM types and different ethnic populations should be performed.
